# Reduction of photobleaching effects in photoacoustic imaging using noise agnostic, platform-flexible deep-learning methods

**DOI:** 10.1117/1.JBO.30.S3.S34102

**Published:** 2025-05-28

**Authors:** Avijit Paul, Christopher Nguyen, Tayyaba Hasan, Srivalleesha Mallidi

**Affiliations:** a Tufts University, Department of Biomedical Engineering, Medford, Massachusetts, United States; b Massachusetts General Hospital, Harvard Medical School, Boston, Massachusetts, United States

**Keywords:** photobleaching, platform-flexible generative deep learning, photoacoustic imaging, acoustic resolution photoacoustic microscopy

## Abstract

**Significance:**

Molecular photoacoustic (PA) imaging with exogenous dyes faces a significant challenge due to the photobleaching of the dye that can compromise tissue visualization, particularly in 3D imaging. Addressing this limitation can revolutionize the field by enabling safer, more reliable imaging and improve real-time visualization, quantitative analysis, and clinical decision-making in various molecular PA imaging applications such as image-guided surgeries.

**Aim:**

We tackle photobleaching in molecular PA imaging by introducing a platform-flexible deep learning framework that enhances SNR from single-laser pulse data, preserving contrast and signal integrity without requiring averaging of signals from multiple laser pulses.

**Approach:**

The generative deep learning network was trained with an LED-illuminated PA image dataset and tested on acoustic resolution PA microscopy images obtained with single-laser pulse illumination. *In vitro* and *ex vivo* samples were first tested for demonstrating SNR improvement, and then, a 3D-scanning experiment with an ICG-filled tube was conducted to depict the usability of the technique in reducing the impact of photobleaching during PA imaging.

**Results:**

Our generative deep learning model outperformed traditional nonlearning, filter-based algorithms and the U-Net deep learning network when tested with *in vitro* and *ex vivo* single pulse-illuminated images, showing superior performance in terms of signal-to-noise ratio (93.54±6.07, and 92.77±10.74 compared with 86.35±3.97, and 84.52±11.82 with U-Net for kidney, and tumor, respectively) and contrast-to-noise ratio (11.82±4.42, and 9.9±4.41 compared with 7.59±0.82, and 6.82±2.12 with U-Net for kidney, and tumor respectively). The use of cGAN with single-pulse rapid imaging has the potential to prevent photobleaching (9.51±3.69% with cGAN, and 35.14±5.38% with long-time laser exposure by averaging 30 pulses), enabling accurate, quantitative imaging suitable for real-time implementation, and improved clinical decision support.

**Conclusions:**

We demonstrate the potential of a platform-flexible generative deep learning–based approach to mitigate the effects of photobleaching in PA imaging by enhancing signal-to-noise ratio from single pulse-illuminated data, thereby improving image quality and preserving contrast in real time.

## 1 Introduction

Photobleaching is the irreversible degradation of a dye molecule, triggered by photon absorption from a rare short-lived excited singlet state or a more common long-lived triplet state after intersystem crossing.^1^^–^^3^ This phenomenon is influenced by intrinsic molecular properties as well as environmental factors such as oxygen concentration, pH, and temperature. As photobleaching occurs with prolonged light exposure, it limits the time window for obtaining quantitative images in optical imaging.^4^^–^^6^ For example, in contrast-enhanced optical image–guided surgical interventions, surgeons may need to frequently readminister or reapply imaging agents, disrupting the procedure. The signal intensity loss due to photobleaching compromises real-time visualization of critical structures during surgery, such as tissues, vessels, or tumors, ultimately leading to diminished sensitivity in detecting subtle anatomical or pathological features. Quantitative analysis, such as measuring the concentration of a dye or tissue metabolic activity, relies on consistent signal intensity. Photobleaching introduces variability in optical signal intensity over time, leading to inaccurate measurements and potentially flawed decisions. Alternatively, to compensate for reduced signal intensity, surgeons might increase laser intensity that can cause tissue heating and phototoxic effects on sensitive structures or develop photostable dyes.^7^^–^^10^ Photobleaching is thus a common challenge in optical imaging, particularly photoacoustic (PA) imaging (PAI) due to its high power delivered within the short nanosecond laser pulse, and its impact is particularly significant in applications demanding high spatial and temporal resolution, where consistent image quality and signal reliability are critical.^11^^–^^14^

PAI, an established hybrid biomedical imaging modality combining optical excitation and acoustic detection,^13^^,^^15^ offers unique advantages over traditional clinical imaging techniques such as X-ray,^16^ MRI,^17^ PET,^18^ and OCT^19^ in terms of radiation exposure or spatial and depth resolution for molecular image-guided surgery. By leveraging optical absorption contrast and providing superior spatial and depth resolution (acoustic), PAI has been successfully applied to both endogenous (label-free) and exogenous chromophores.^20^ Among its configurations, acoustic-resolution PA microscopy (AR-PAM^21^) and optical-resolution PA microscopy (OR-PAM^22^) have been widely used for visualizing microscopic structures. AR-PAM achieves resolution by focusing the acoustic transducer, whereas OR-PAM relies on tight optical focusing for superior lateral resolution.^22^^–^^24^ However, AR-PAM systems require 2D raster scanning for image acquisition, during which multiple laser pulses (20 to 30 per scan point) are typically averaged to enhance the signal-to-noise ratio (SNR).^25^ As the laser pulse repetition frequency for a typical Nd-YAG pumped optical parametric oscillator laser is typically low (5 to 20 Hz), this averaging decreases the AR-PAM scan speed and eventually increases the plausibility of prolonged laser exposure which might impose a burden of photobleaching when using exogenous contrast agents such as indocyanine green (ICG). ICG, a commonly used, clinically approved contrast agent,^26^^,^^27^ absorbs photons and undergoes excitation to a singlet state, followed by nonradiative relaxation or interaction with molecular oxygen to generate reactive oxygen species (ROS).^28^^,^^29^ ROS-induced oxidative damage causes structural degradation of ICG, leading to reduced optical absorption and diminished PA signals. This degradation complicates quantitative analyses, such as concentration measurements and monitoring biodistribution, and limits the effective duration for which contrast agents can be used in imaging.

Mitigating photobleaching in PAI involves a trade-off between reducing illumination intensity or exposure time and maintaining adequate SNR for image reconstruction. Low-intensity laser pulses below the photobleaching threshold can be employed, but this results in noisy images that compromise contrast and resolution. Alternative approaches, such as modifying or encapsulating ICG with nanoparticles to enhance photostability^7^^–^^10^ or reducing ROS generation by altering environmental factors,^30^^–^^33^ are technically challenging, lack clinical approval, and require extensive safety and biological feasibility studies before clinical implementation. A promising alternative is to minimize the number of laser pulses per scan point, ideally using single-pulse illumination, to reduce cumulative energy deposition and photobleaching risk. However, single-pulse illumination produces inherently noisy data due to low energy delivery, posing challenges for high-quality image reconstruction.

In this study, we address these challenges by leveraging a platform-flexible generative deep learning (DL) model^34^ to enhance SNR in single-pulse PAI before photobleaching occurs. Traditional algorithms such as Savitzky–Golay (SG),^35^ Wiener,^36^ and BM3D^37^ are not efficient in generating high SNR images, and they require prior estimation of the noise distribution. DL, with its ability to generalize across unseen test data and automatically learn features from training data, has shown significant promise in biomedical imaging.^38^^–^^40^ Specifically, convolutional neural network (CNN)-based architectures,^41^^–^^44^ such as U-Net,^45^^–^^48^ have been widely applied for denoising and image enhancement tasks. Recent work has demonstrated the use of U-Net variants for improving lateral resolution in AR-PAM and denoising low-fluence OR-PAM images.^49^ However, most existing studies focused on enhancing A-line signals before image reconstruction, which requires access to raw data—a limitation in certain PAI systems. To overcome this, our DL framework directly processes noisy images obtained from single-pulse illumination, bypassing the need for A-line access. Although GAN-based^50^ approaches have been explored in various imaging modalities,^51^^–^^54^ their applications to PAI,^48^^,^^55^^–^^58^ particularly for mitigating photobleaching, remain underexplored. It is worth noting that the current work presents several unique advancements specifically designed for our problem domain, demonstrating noise-invariant platform flexibility through an optimized generative model. To validate our approach, we conducted phantom experiments with ICG solutions, where we observed a decline in PA amplitude with multiple pulse illuminations. Our cGAN model successfully reconstructed high-quality SNR images from single-pulse noisy data, offering a robust solution to the dual challenges of photobleaching and noisy imaging in real-time PAI. Furthermore, developing platform-independent DL architectures is vital for robust biomedical imaging. Such models can handle diverse input data and ensure consistent performance across systems. In PAI, platform flexibility minimizes reliance on specific hardware and maintains high SNR across configurations. Despite its significance, platform-flexible DL networks for PAI remain underexplored. Platform flexibility in this work refers to the model’s ability to generalize across different noise characteristics inherent to LED-based and AR-PAM imaging systems. This flexibility ensures robust denoising performance without requiring retraining or fine-tuning for each platform. Using a custom loss function-based cGAN trained on AcousticX LED-illuminated data and tested on AR-PAM data, we demonstrate platform flexibility for SNR improvement, paving the way for broader DL adoption in diverse PAI applications.

## 2 Methodology

### 2.1 Microscopic Imaging System

To perform PA imaging, the AR-PAM system employed an OPO (Phocus HE Benchtop, OPOTEK, Carlsbad, California, United States) to generate pulsed laser energy. The subsequent acoustic signal generated was collected using a 1-in. focused transducer (V324-SU, Olympus, Tokyo, Japan) with a center frequency of 25 MHz. This signal was amplified by a pulser/receiver (DPR500, JSR Ultrasonics, Pittsford, New York, United States), which also facilitated signal digitization by a data acquisition card (CSE161G2, GaGe, Ferndale, Michigan, United States).

To transmit the laser energy to the sample, the system utilized an achromatic doublet (AC254-030-B-ML, Thorlabs, Newton, New Jersey, United States) and an aspheric condenser (ACL2520U-B, Thorlabs) to couple the laser beam from the OPO fiber into a secondary fiber bundle. This secondary fiber bundle consisted of seven multimode fibers (FT1000EMT, Thorlabs) epoxied together toward the proximal end for coupling. The distal end of the fiber bundle was designed in a fanned configuration and connected to a 3D-printed transducer-and-fiber mount, which oriented the fibers to mimic an optical condenser.

### 2.2 Nonlearning Traditional Models

We implemented several traditional noise removal algorithms such as SG, Wiener, and BM3D to compare with the cGAN model. For the SG noise removal algorithm, local polynomial fitting ensures smoothness without significant loss of signal shape. Wiener filter aims at minimizing the mean square error between the original and estimated signals. It adapts based on local signal statistics, resulting in a trade-off between smoothness and signal preservation. However, this filter is ideal when we have prior knowledge about both the signal and noise characteristics, such as their power spectra. BM3D is another state-of-the-art denoising algorithm that groups similar patches from an image and performs 3D transformation to filter the noise. The key advantage of this filter is the ability to group similar image blocks and process them together in 3D space, allowing it to outperform simpler denoising techniques in terms of detail preservation and noise reduction. In the SG algorithm, we employed a third-order SG filter (sgolayfilt in MATLAB^®^) with a frame length of 9 for convolution-based smoothing.^59^ After several hits and trials, we observed that a third-order SG filter with a frame length of 9 was used to preserve signal characteristics while reducing noise. The third-order polynomial enables the filter to preserve both linear and quadratic signal trends, as well as subtle curvature variations, which are common in PA signals (e.g., vascular structures or temporal dynamics). Higher-order filters might be overfitted to noise, introducing artifacts, whereas lower-order filters (e.g., first or second order) might over-smooth, erasing critical features like sharp edges or peaks. We observed that the frame length of 9 balances noise suppression and feature preservation. It is to be noted that larger frame lengths might provide stronger noise suppression but risk smoothing out high-frequency details, whereas smaller frame lengths retain more detail but may inadequately suppress noise.^60^^,^^61^ The Wiener algorithm^36^^,^^62^ was implemented using a pixel-wise adaptive low-pass filter (wiener2 function in MATLAB^®^) with a neighborhood size of 5×5 to estimate the local image mean and standard deviation. Multiple trials with several neighborhood window compositions indicate that the choice of a 5×5 neighborhood for the Wiener filter in PA imaging is grounded in its ability to provide a balanced trade-off between noise suppression, feature preservation, computational efficiency, and compatibility with the spatial resolution of PA systems. In BM3D, we initially estimated the standard deviation (σ) using a multichannel approach and subsequently applied Python’s bm3d function with the estimated σ and BM3DStages.ALL STAGES configuration. This configuration, which includes Wiener filtering in addition to hard thresholding, provides slightly improved performance for our system-specific noise compared to other BM3DStages.

### 2.3 Deep Learning Architectures

#### 2.3.1 U-Net

Within our implementation, we employed two DL networks, one of which was the original U-Net architecture. U-Net, a convolutional neural network (CNN)-based architecture, follows an encoder–decoder structure, where the encoder (contracting path) captures context and reduces spatial dimensions through successive layers of convolution, ReLU activation, and max-pooling operations.^63^ This is followed by a decoder (expanding path) that uses up-convolutions (transposed convolutions) to restore spatial resolution. At each step of up-sampling, skip connections from the corresponding layers of the encoder are concatenated to the decoder, enabling the model to retain fine-grained information from earlier stages. These skip connections help U-Net preserve spatial details while improving localization, making it highly effective for tasks like biomedical imaging. The network’s symmetry and ability to work with limited training data are key strengths. We utilized the “mean absolute error” loss function and the “Adam” optimizer with an initial learning rate of $1\times 10^{-4}$. The U-Net is distinguished by its inclusion of residual skip connections, which contribute to the network’s notable effectiveness. For a more comprehensive understanding of the U-Net’s impact on PA imaging, please refer to Ref. 64.

#### 2.3.2 Conditional GAN

**Fig. 1:**
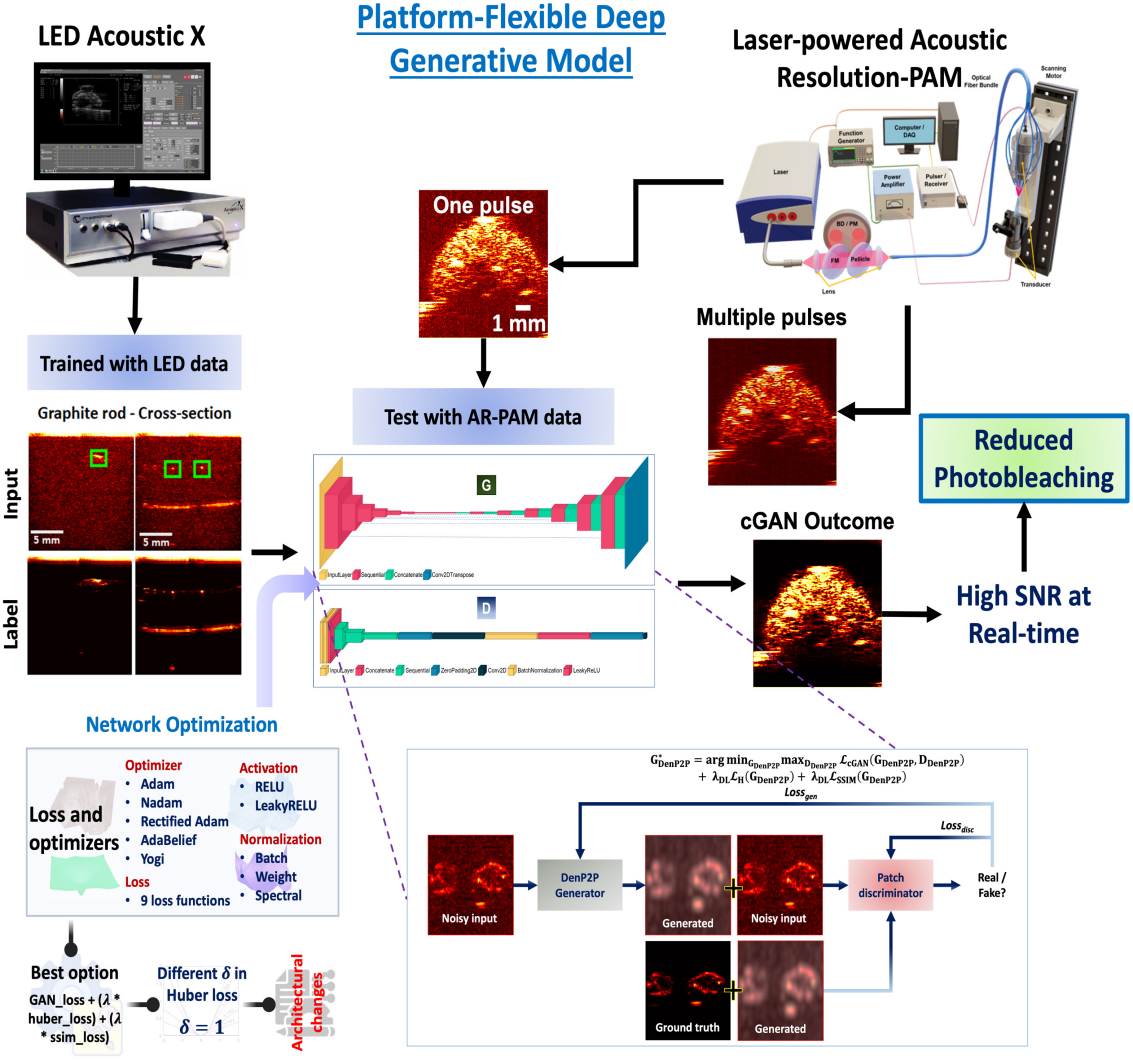
Platform-flexible cGAN workflow where training was done with LED-illuminated PAI data and tested with AR-PAM data. G, generator; D, PatchGAN discriminator where layer depictions were generated by the visualkeras library. The network optimization modules are shown at the left bottom portion where we implemented several loss functions, optimizers, activation layer, and architectural changes.

In our implementation, we devised a custom loss function based Pix2Pix network (cGAN denoising model); the process schematic is illustrated in Fig. 1. The general workflow of our cGAN training with AcousticX LED data and testing with AR-PAM data is shown in the figure. G and D showcase the architecture of the generator and the PatchGAN discriminator, respectively. The extended inset portrays the functioning of the cGAN with our custom loss function.

##### Generator

We employed a U-Net-based model as the generator. The down-sampling and up-sampling operations are accompanied by corresponding residual connections which help recover local information lost during the down-sampling process. Each block in the down-sampling pathway consists of a series of convolution, batch normalization, and rectified linear unit (RELU) activation layers. The first block does not include a batch normalization layer, and the first three blocks do not have a dropout layer. Similarly, each block in the decoder section is comprised of a sequence of transposed convolution, batch normalization, and RELU layers, with the last three blocks excluding a dropout layer.

All dropout layers have a probability of 0.5 for discarding the random nodes. In addition, the weight initialization for all layers was performed using “*he_normal*,” which draws weights from a truncated normal distribution. This initialization strategy works well with RELU activation due to its controlled weight initialization, facilitating the rapid convergence to a global minimum of the loss function.

##### Discriminator

We employed a convolutional PatchGAN classifier in our implementation. This classifier focuses on classifying a 70×70 portion of the input image based on a 30×30 output image patch. Each block of the discriminator consists of a convolution, batch normalization, and ReLU layer. However, the first block does not include a normalization layer and the last layer has a leakyReLU activation instead of a regular ReLU activation for better gradient flow, avoiding dead neurons, and stabilizing the training while effectively capturing local texture details.

##### Generator and discriminator objectives

The cGAN computational architecture attempts to find a mapping from the observed image (noisy input) x and random noise vector z to label (ground truth) y, G:{x,z}→y. The generator of the cGAN learns a structured loss by minimizing the difference between the network outputs and the corresponding targeted images. The cost function of a cGAN is normally expressed as $L_{c\mathrm{GAN}}(G,D)=E_{x,y}[\log\;D(x,y)]+E_{x,z}[\log(1-D(x,G(x,z))]$, which represents a two-player game between generator G and discriminator D where G tries to maximize the objective against the antagonist D. Hence, the generator’s objective function is expressed as $G^{*}=\arg\;\min_{G}\;\max_{D}\;L_{c\mathrm{GAN}}(G,D)$. Keeping the loss function of the discriminator unchanged from the Pix2Pix model, we used Huber loss, (instead of L1, mean absolute error, loss) and an additional loss, structural similarity (SSIM) loss for the generator. As a result, G attempts to fool D as it tries to produce images that are not distant from the real data following the Huber loss, which considers both $L_{1}$ and $L_{2}$ norm based on a δ value and are structurally similar to the ground truth based on the SSIM loss. The Huber loss^65^^,^^66^ is defined as

$$L_{h}(G)=\{\begin{array}{ll} \frac{1}{2}(y-G(x,z){)}^{2} & |y-G(x,z)|\leq \delta \\ \delta \cdot [{\left\|y-G(x,z)\right\|}_{1}-\frac{1}{2}\delta ] & \text{otherwise} \end{array}$$

and SSIM loss is defined as $L_{\mathrm{SSIM}}(G)=\frac{1}{N}\sum 1-\mathrm{SSIM}(y,G(x,z))$ where SSIM(A,B) is defined in Ref. 67. These additional losses help the cGAN resist the vanishing gradient problem because the loss metrics can measure how far the generated data distribution is from real ground truth, and our generator learns from discriminator losses. SSIM loss^68^ computes localized statistics (mean, variance, etc.) in image patches, ensuring nonzero gradients even in flat regions where pixel-wise losses such as MSE may fail. By emphasizing structural patterns and textures rather than individual pixel intensities, SSIM loss maintains gradient flow and improves the network’s ability to capture high-level image features. The combined generator loss functions give us the final objective function of G as follows:

$$G^{*}=\arg\;\min_{G}\;\max_{D}\;L_{c\mathrm{GAN}}(G,D)+\lambda_{DL}L_{h}(G)+\lambda_{DL}L_{\mathrm{SSIM}}(G),$$

where $\lambda_{DL}$ is the regularization parameter with a value that was chosen after multiple trials.

The Adam optimizer^69^^,^^70^ was utilized for both the generator and discriminator, with an initial learning rate of 0.0001. The parameters $\beta_{1}$ and $\beta_{2}$ were set to 0.5 and 0.999, respectively, and ∈ was set to $10^{-7}$. The parameters ∈, $\beta_{1}$, and $\beta_{2}$ in the Adam optimizer play distinct roles in controlling how the algorithm updates weights during training. ∈ is a small constant added to the denominator for numerical stability in the learning process, particularly when gradients are very small. $\beta_{1}$ is the exponential decay rate for the first moment estimate (mean of the gradients), which controls how quickly the moving average of the gradients adapts to new gradient information. $\beta_{2}$ is the exponential decay rate for the second moment estimate (variance of the gradients), which controls how the moving average of the squared gradients adapts. During training, a batch size of 1 was employed and the process consisted of 40,000 steps that allowed the network to focus on the unique structural and pixel-level details of each image pair, enhancing fine-grained outputs (instance normalization).^71^^,^^72^ This approach is crucial for preserving subtle features in high-resolution tasks. Although larger batch sizes improve convergence, they average gradients across samples, potentially diluting unique contributions and reducing training diversity. Moreover, smaller batch size introduces more stochasticity, helping the discriminator generalize better, avoid overfitting, and stabilize optimization by escaping poor local minima. Within the combined generator objective function, the regularization parameter $\lambda_{DL}$ was set to 100. In addition, the δ parameter for the Huber loss was set to 1. After systematically evaluating a range of generator loss functions (combinations of L1, L2, Huber, SSIM, and PSNR losses) and optimization methods (Adam, Nadam, Rectified Adam, AdaBelief, and Yogi),^73^ Adam emerged as the preferred optimizer when combined with the original GAN, Huber, and SSIM losses, due to its shorter training time (93 s per 1000 steps) and robust generalization, despite no statistically significant differences in image quality metrics across optimizers. In addition, we optimized the delta (δ) value for the Huber loss function, identifying δ=1 as the optimal setting. Further experimentation with activation functions (ReLU and Leaky ReLU) and normalization layers (batch, weight, and spectral)^74^ led to the selection of ReLU activation and batch normalization as the most effective combination (see bottom left part of Fig. 1). The detailed optimization and tuning of the hyperparameters (nine different loss functions and delta parameter of huber loss) and architectural modifications (activation layer and normalization strategies) are provided in Tables S1, S2, and S3 in the Supplementary Material.

All DL codes were implemented using TensorFlow (version 2.9.2) with the Keras backend and executed on the Google Colab Pro platform. The computations were performed on a Tesla T4 GPU (CUDA version: 11.2) with 25.45 GB of RAM. The training process took ∼93 to 94 s per 1000 steps.

### 2.4 Database

#### 2.4.1 Training data

Frame averages impact image quality: fewer averages yield low-SNR, noisy images, whereas higher averages provide high-SNR images. Using the LED-based AcousticX PA system, we captured 800 paired images: low-SNR inputs (128 frame averages) and high-SNR labels (25,600 frame averages). There was no spatial misalignment between inputs and labels as the transducer remained stationary, and the noise was system-generated (e.g., shot and thermal noise). The training data came from a different imaging platform, handling macro-scale objects, unlike the AR-PAM system, which focuses on microscopic data.

#### 2.4.2 Test data

We conducted test data collection using our in-house built AR-PAM system. For *in vitro and ex vivo* imaging samples, we utilized carbon fiber, mouse kidney, and mouse tumor specimens. To investigate the photobleaching phenomenon, we conducted an experiment using a polyethylene tube filled with a mixture of 0.5-μM ICG and deionized water, which was securely positioned at both ends of a box. To assess the plausibility of inducing photobleaching of ICG by laser pulses, we illuminated the tube at a single point for 15 min in three separate trials using both deionized water and dimethyl sulfoxide. In all cases, we observed a consistent decrease in the PA amplitude over time, indicating the occurrence of photobleaching. Subsequently, we performed a 2D scanning of the tube in the x−z direction with a step size of 100  μm. Two laser pulse illuminations were employed: single-pulse illumination (no averaging) and 30-pulse illumination.

### 2.5 Image Quality Metrices

To check the quality of the cGAN-generated output images, we used three important reference image quality metrics.

#### 2.5.1 SNR

We used SNR as a no-reference quality metric^75^ measured in arbitrary units to accommodate signals with a wide dynamic range. It is defined as $\mathrm{SNR}=\frac{\mu_{\mathrm{ROI}}}{\sigma_{BG}}$, where $\mu_{\mathrm{ROI}}$ is the mean signal amplitude of the target region of interest (ROI), and $\sigma_{BG}$ is the standard deviation of the background noisy region.

#### 2.5.2 CNR

Contrast to noise ratio (CNR)^76^ is a no-reference image quality metric, which compares the standard deviation of the target ROI with respect to the background noisy region. It is defined as $\mathrm{CNR}=\frac{\sigma_{\mathrm{ROI}}}{\sigma_{BG}}$, where $\sigma_{\mathrm{ROI}}$ is the standard deviation of the target ROI and $\sigma_{BG}$ is the standard deviation of the background noisy region.

#### 2.5.3 GCNR

Generalized CNR^77^^,^^78^ is a quantitative measure of how distinguishable two ROIs are in an image, accounting for overlap in their intensity distributions by combining the effect of contrast and noise. Unlike metrics that rely purely on absolute intensity differences, GCNR evaluates the separability of the distributions, making it robust to noise and intensity variations. GCNR is computed as $\mathrm{GCNR}=1-\int \min(p_{1}(I),p_{2}(I))dI$, where $p_{1}(I)$ and $p_{2}(I)$ are the probability density functions of the pixel intensities in the two ROIs, and I is the intensity value.

## 3 Results and Discussion

**Fig. 2:**
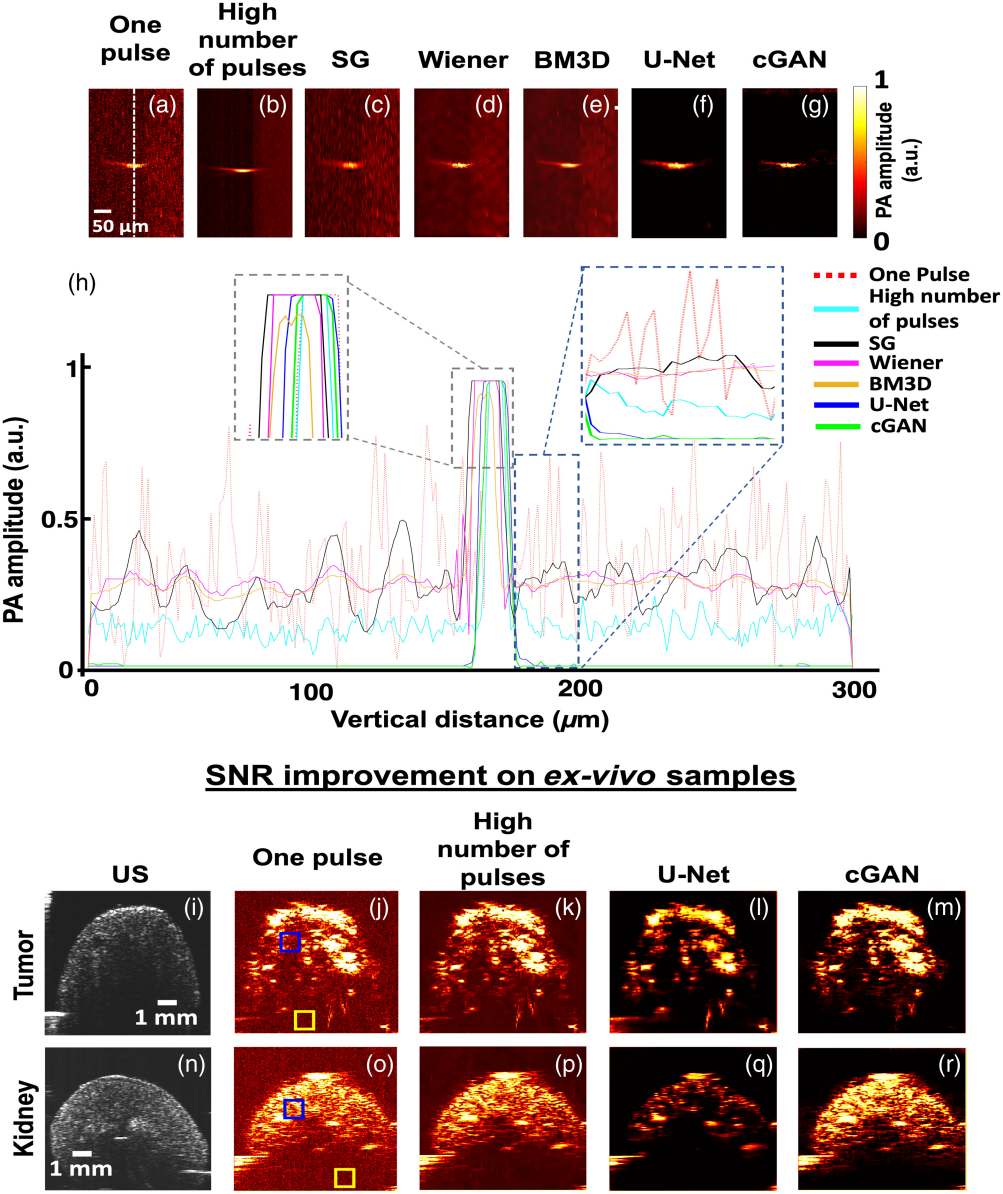
(a)–(g) Comparison between nonlearning traditional denoising algorithms with our cGAN DL model. The bottom graph (h) shows the PA amplitude along the axial direction depicted by a white dotted line for one pulse illumination (red), high number of pulses (cyan), SG (black), Wiener (magenta), BM3D (yellow), U-Net (blue), and our cGAN (green). The left inset is the enlarged version of the peak signal and the right one shows the zoomed-in version of the background noise. (i)–(r) US and PA images for *ex vivo* samples produced with one laser pulse, average of 20 laser pulses, U-Net, and cGAN methods.

To demonstrate the efficacy of our DL model for improving the SNR and CNR, we imaged a carbon fiber with two laser pulse illuminations: one pulse (input) and 20 pulse averaging (ground truth). In Fig. 2, cross-section images of the carbon fiber are shown for one pulse [Fig. 2(a)] and average of 20 pulses [Fig. 2(b)]. Noise removal filters were applied to the one pulse images using traditional nonlearning algorithms [SG: Fig. 2(c), Wiener: Fig. 2(d), and BM3D: Fig. 2(e)], U-Net [Fig. 2(f)] and our Pix2Pix-based cGAN denoising model [Fig. 2(g)]. Figure 2(h) shows the line profile of the one pulse illumination (red line), a high number of pulses illumination (cyan), SG noise removal (black), Wiener filter (magenta), BM3D (yellow), U-Net image (blue), and the cGAN generated image (green) for the carbon fiber along the axial direction. The left inset in Fig. 2(h) shows the enlarged display of the PA peak signal amplitude and the right enlarged inset denotes the background region profile. For *ex vivo* samples, we utilized mouse kidney and tumor data as shown in Figs. 2(i)–2(m) and Figs. 2(n)–2(r), respectively. The ultrasound (US) images accompanying each row provide morphological confirmation of the biological structures. For quantitative analysis, the blue square was selected as the ROI to calculate image quality metrics such as SNR and CNR. The yellow box served as the background to assess how well each method suppressed noise in non-ROI areas. The image quality metrices for *ex vivo* samples are depicted in Table 1.

**Table 1:** Image quality metrices for different traditional and DL denoising methods.

Imaging process	SNR	CNR	GCNR
***Ex vivo*** **sample: Tumor**			
One pulse	73.75 ± 6.32	4.12 ± 2.16	0.734 ± 0.016
High number of pulses	79.81 ± 3.61	7.68 ± 1.29	0.981 ± 0.014
SG	77.01 ± 6.17	5.43 ± 1.47	0.787 ± 0.021
Wiener	75.4 ± 4.74	5.62 ± 2.54	0.791 ± 0.015
BM3D	77.93 ± 8.37	6.32 ± 2.88	0.834 ± 0.014
U-Net	84.52 ± 11.82	6.82 ± 2.12	0.861 ± 0.013
Pix2Pix cGAN	**92.77 ± 10.74**	**9.9 ± 4.41**	**0.979 ± 0.011**
***Ex vivo*** **sample: Kidney**			
One pulse	72.76 ± 9.25	6.19 ± 4.36	0.739 ± 0.018
High number of pulses	82.11 ± 4.19	7.53 ± 1.96	0.987 ± 0.019
SG	78.09 ± 3.62	7.29 ± 3.79	0.807 ± 0.019
Wiener	81.34 ± 6.49	6.41 ± 2.46	0.793 ± 0.014
BM3D	78.81 ± 7.89	6.98 ± 3.44	0.814 ± 0.012
U-Net	86.35 ± 3.97	7.59 ± 0.82	0.851 ± 0.015
Pix2Pix cGAN	**93.54 ± 6.07**	**11.82 ± 4.42**	**0.983 ± 0.012**

Each of the corresponding table entries is depicted as mean ± standard deviation.

Traditional nonlearning filters demonstrated a moderate ability to reduce noise, but they fell short in terms of preserving structural details. The SG filter marginally smoothed the background noise but oversimplified the sharp features, whereas the Wiener and BM3D filters introduced varying degrees of blurring. By contrast, the DL-based approaches, especially the Pix2Pix-based cGAN model, achieved superior performance by preserving fine details and maintaining the integrity of the axial line profiles. This can be seen in Fig. 2(h), where the cGAN’s line profile closely follows that of the images acquired with a high number of pulses across both the PA peak signal region and background areas. The traditional noise filters’ line profiles are less noisy than the single-pulse data but significantly noisy in the background regions compared with the DL methods. The *ex vivo* results reinforced the carbon fiber experiment’s observations where our cGAN model (fourth column) outperforms the U-Net in preserving the detailed structure of the tumor while effectively denoising the images, resulting in higher-quality images. Multipulse illumination significantly improved image clarity but required longer acquisition times, which might lead to undesirable effects of photobleaching and phototoxicity. Both DL models performed well in reducing noise, but the U-Net’s tendency to blur finer details led to the loss of critical anatomical information. This was evident in the kidney and tumor’s more delicate structures, where the Pix2Pix-based cGAN outperformed the U-Net by offering clearer, more detailed visualizations without sacrificing noise suppression, as also evident from Table 1. Our study presents a real-time, single-pulse denoising framework designed for applications such as PDT and *in vivo* imaging, where multiframe averaging is impractical due to motion artifacts, increased laser exposure, and photobleaching risks. Although averaging improves GCNR by reducing contrast fluctuations, it is not feasible in real-time settings. Our method enhances image quality at the single-pulse level, achieving significant improvements in SNR and CNR, which are also clinically relevant for preserving fine structural details. Unlike GCNR, which focuses on separability, SNR and CNR impact the visibility of fine structures, making them useful for clinical use. In addition, PA image noise is spatially and spectrally nonuniform, making GCNR sensitive to residual structured noise. Our spatially adaptive noise suppression balances contrast enhancement with feature preservation, ensuring superior perceptual image quality. Unlike post-processing techniques that enhance global contrast at the cost of fine-edge details, our method retains crucial structures essential for clinical interpretation.

**Fig. 3:**
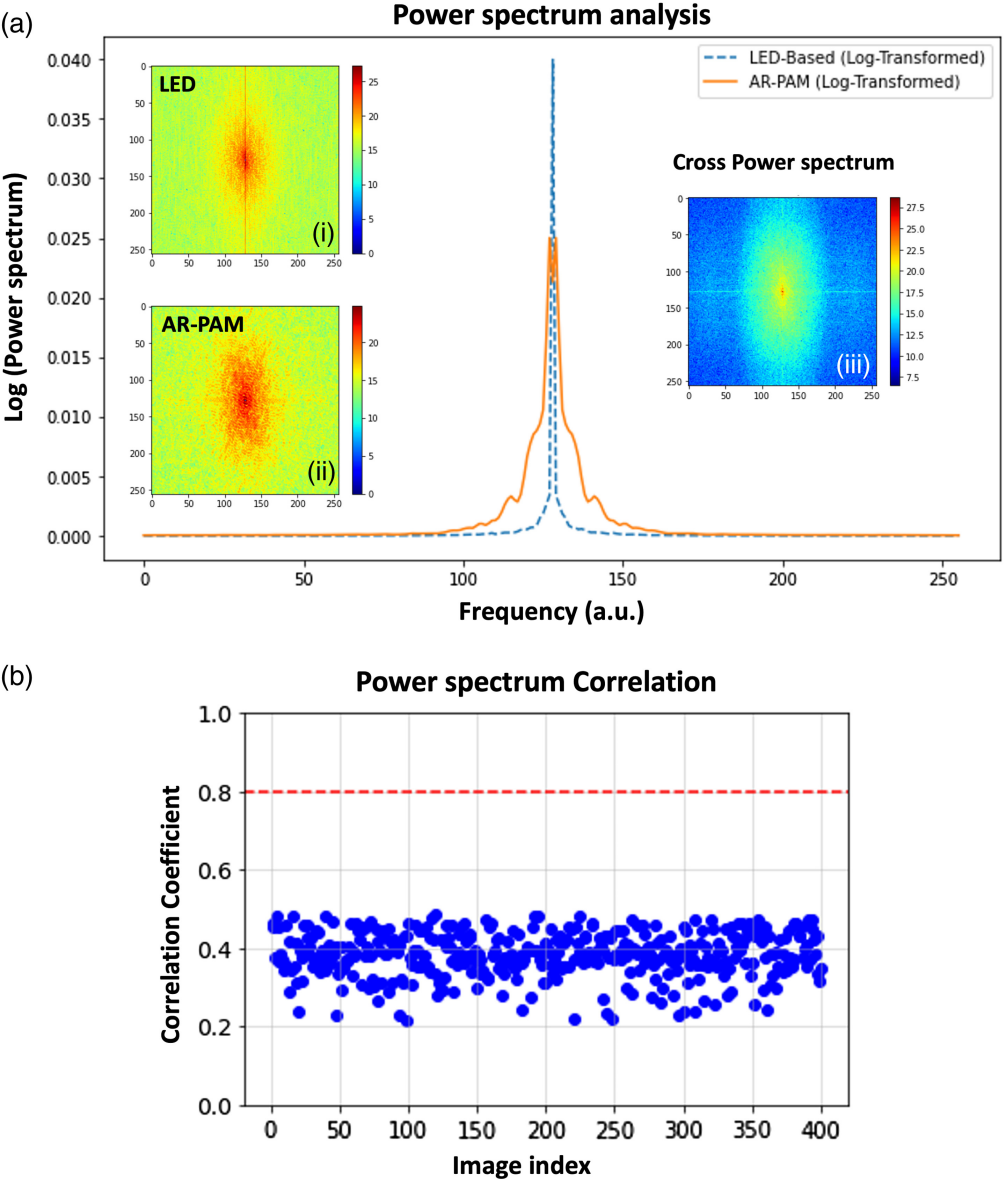
Characterization of noise profiles across LED and AR-PAM imaging platforms. (a) Normalized power spectrum analysis for LED and AR-PAM systems, calculated as the log-transformed squared magnitude of the FFT to compress the dynamic range. Insets (i) and (ii) show the spatial frequency content of the noise for the two platforms, highlighting differences in noise profiles. Inset (iii) presents the cross-power spectrum, identifying frequency regions where the noise profiles exhibit similarities or differences. (b) The cross-power spectrum highlights notable differences, particularly in the low-frequency regions. Correlation analysis of the flattened power spectra reveals a low correlation coefficient (<0.8), confirming significant dissimilarity in the noise profiles.

To substantiate the claim of platform flexibility with respect to noise distribution across different imaging platforms, we first characterized the noise profiles (statistical and spectral properties of noise^79^^–^^81^) of both LED and AR-PAM systems using noise power spectrum and correlation analysis for spatial noise statistics. Figure 3(a) depicts the power spectrum analysis for LED and AR-PAM inputs, which is calculated as the log-transformed of the squared magnitude of the FFT so that the dynamic range is compressed. The spectra are then normalized to their maximum values for comparison. The inset figures [Figs. 3(a,i,ii)] show the spatial frequency content of the noise for the two imaging platforms, highlighting differences between them. The inset in Fig. 3(a,iii) analyzes the cross-power spectrum to study relationships between the noise profiles, which identify frequency regions where the noise profiles are similar or different. Finally, we quantify the similarity between two systems’ noise profile distributions by calculating correlation coefficient^82^^–^^84^ between the two power spectra flattened to one dimension [Fig. 3(b)]. The LED spectrum has a higher amplitude at low frequencies than AR-PAM, whereas some of the higher frequency zones of AR-PAM data has higher amplitude compared with LED-based outcomes. The cross-power spectrum shows a notable difference in the low-frequency zones. Different amplitude values at the same frequency locations in the power spectra for LED and AR-PAM data suggest that the noise profiles or signal intensities vary between the two platforms. The correlation analysis between the two spectra is significantly <0.8, which also indicates high dissimilarity in their noise profiles.

**Fig. 4:**
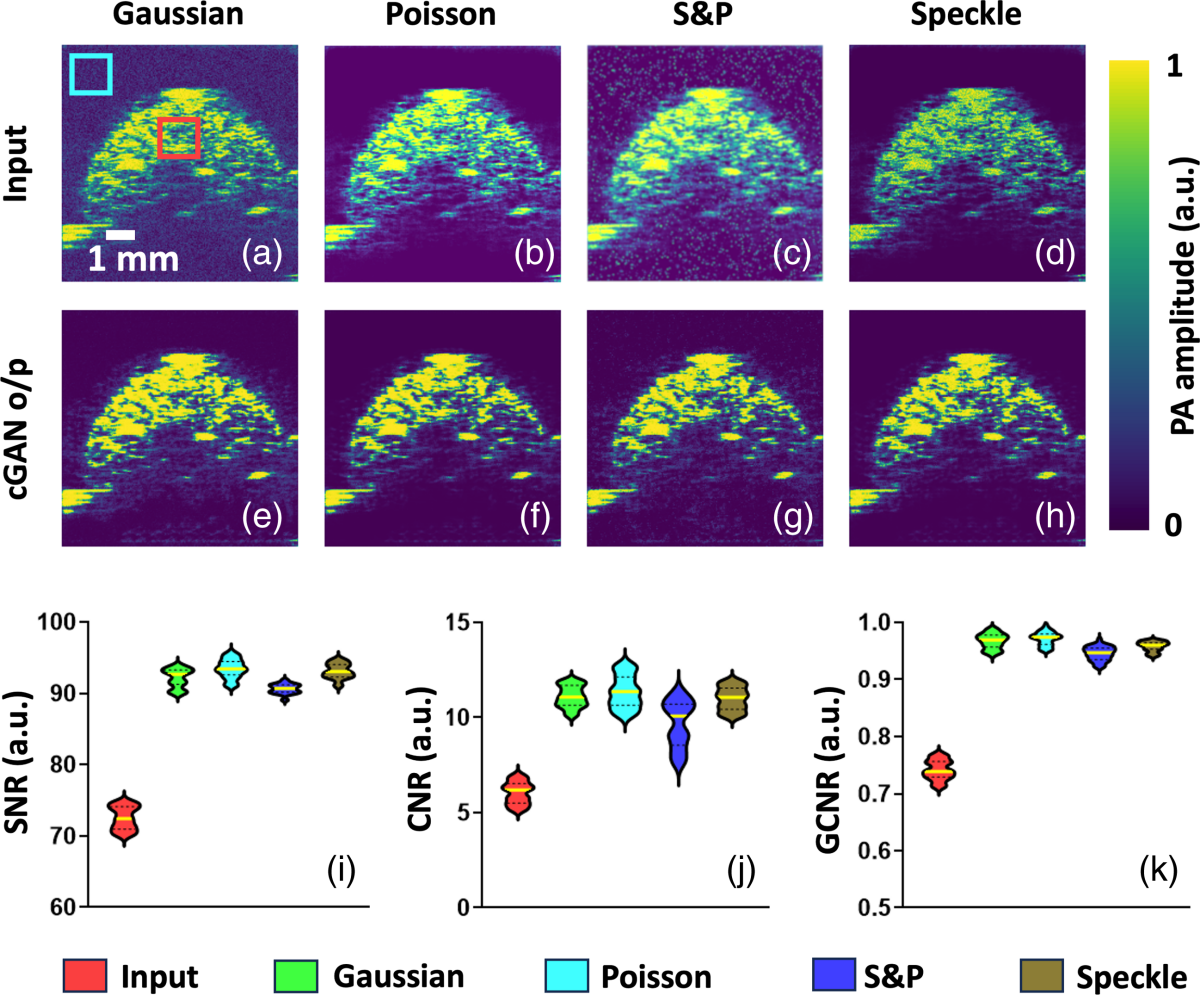
Validation of the model’s flexibility and robustness to diverse noise conditions. (a)–(h) Denoising performance on AR-PAM high-pulse-averaged data with synthetically injected Gaussian, Poisson, S&P, and speckle noise. (i)–(k) Violin plots of image quality metrics (e.g., SNR, CNR, and GCNR) across datasets with varying noise profiles, showcasing the model’s noise-invariance capabilities. The two sample regions are denoted by red and cyan colored boxes where one has PA signal contents and another one does not have them.

For further validation on flexibility, our next approach was to synthetically inject Gaussian (σ=0.1),^85^^–^^88^ Poisson,^86^^,^^88^^,^^89^ salt and pepper (S&P) with 10% for pixel destruction^90^^,^^91^ and speckle noise (uniform distribution having zero mean and 0.1 as variance)^86^^,^^88^^,^^92^ into the AR-PAM high number of pulses averaged data to simulate diverse noise conditions. Visual validation from Figs. 4(a)–4(h) corroborates that our DL model consistently achieved high denoising performance, indicating its robustness to noise variations. The three violin plots in Fig. 4(i)–4(k) depicting image quality metrics (SNR, CNR, and GCNR) analysis across datasets with diverse noise profiles evidently suggest our model’s noise-invariance capabilities. In addition, we implemented several Gaussian noise profiles with different variances [Figs. S1(a)–S1(f) in the Supplementary Material] as mentioned in the top right corner of the corresponding input images. We provided a visual depiction of our model’s outcomes in the second row [Fig. S1(g-l) in the Supplementary Material] demonstrating how our model performs under increasing levels of injected noise. This visually confirms the model’s robustness up to a certain noise threshold. We also reported quantitative image quality metrics under varying noise levels in the third row [Figs. S1(m)–S1(o) in the Supplementary Material], showing how performance degrades as noise variance increases. Overall, our results confirm that although extreme noise levels do affect the model, they maintain satisfactory performance within practical noise limits encountered in PAI. Future work will focus on validating the model’s noise invariance across additional platforms (e.g., MSOT or handheld devices) and exploring domain adaptation techniques to further enhance its generalizability to systems with varying noise characteristics.

**Fig. 5:**
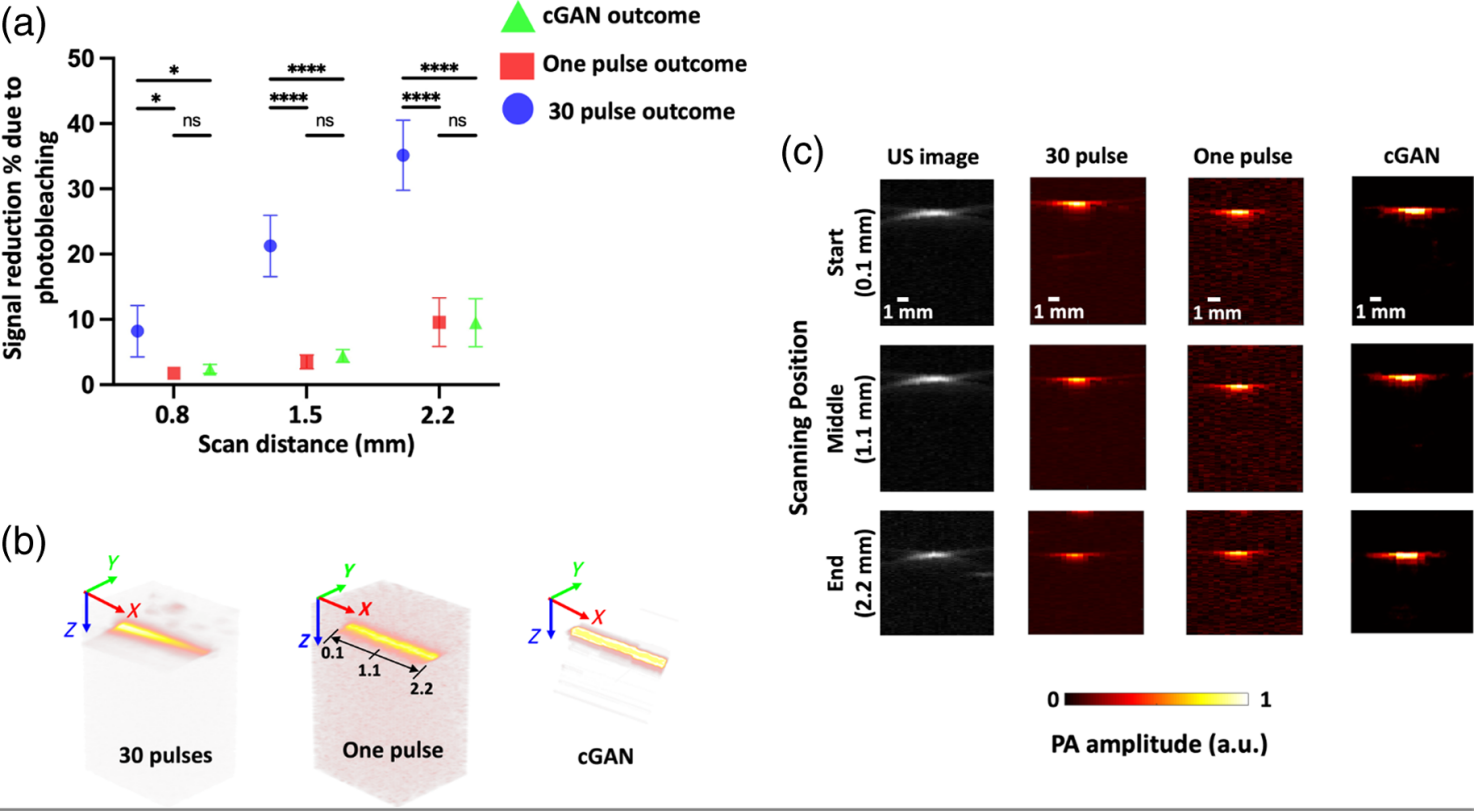
Comparison of photobleaching experiment of ICG. (a) Percentage of ICG photobleaching at different scanning positions within the tube for one pulse, high number of pulses (30), and cGAN denoising image obtained with single pulse illumination. Photobleaching is considerably more pronounced at the tube’s end position compared to the initial scanning position. We performed a two-way ANOVA to compare the results within each method across the different scanning distances. The analysis reveals that photobleaching becomes more pronounced as the scanning distance increases. Comparing the different imaging procedures (single pulse, 30-pulse illumination, and cGAN) across all scanning distances (especially 1.8 and 2.2 mm), we found that the 30-pulse illumination caused significantly more photobleaching than both the single pulse and cGAN methods. ns, not significant, *p<0.05, ****p<0.0001. (b) Volumetric 3D images of the tube showing a comparison between one-pulse and 30-pulse illuminated PA images. (c) Comparison of PA amplitude at three scan positions for one pulse, high number of pulses (30), and cGAN denoising. The left most column depicts the ultrasound images of the corresponding scanning positions of the tube.

To observe the photobleaching effect of contrast agents used for PAI, we initially illuminated the tube filled with ICG dissolved in deionized water at a specific scanning location for three trials, each for 15 min. The PA signal amplitude consistently reduced by ∼35% across all trials, pointing to significant photobleaching. The uniformity of this reduction strongly suggests that water-based ICG solutions are prone to photobleaching under prolonged laser exposure, which can compromise the accuracy and consistency of PA imaging. The fact that photobleaching occurs uniformly indicates that the reduction in signal is not localized but rather affects the entire imaging area. This can lead to a decrease in sensitivity and accuracy in detecting and quantifying contrast-agent-labeled structures, especially in applications such as longitudinal studies where signal consistency is paramount. To visually demonstrate these effects, a 2D raster scan of tubes filled with ICG and deionized water with a step size of 100  μm is shown in Fig. 5. Figure 5(a) illustrates the calculated percentage of photobleaching or photodamage of ICG within the tube at four distinct cross-sections spaced 0.7 mm apart. The percentage of photobleaching was determined by comparing the PA amplitude values at corresponding locations to the initial starting position. Note that the initial starting position was at 0.1 mm and therefore shows no photobleaching. Thirty-pulse illumination imaging at 2.25-mm scanning distance shows significant photobleaching % (35.14±5.38%) compared with single pulse imaging (9.58±3.74%) and our cGAN derived imaging (9.51±3.69%). Although the current results are derived from retrospective data analysis, the computational speed of the cGAN model (<27  ms per frame) demonstrates its potential for real-time applications as the use of a single-pulse rapid imaging technique minimizes the need for long exposure inherently reducing photobleaching. To further validate this, we plan to conduct experiments integrating the model with a live imaging setup in future work.

Figure 5(b) showcases volumetric images obtained using one pulse and 30 laser pulse illumination. Individual B-scans were obtained in the Y direction, orthogonal to the tube’s diameter. A $2\times 3\;{\mathrm{mm}}^{2}$ area of the tube was scanned, consisting of 30 cross-sections spaced 100  μm apart. Figure 5(c) presents three of those cross-sections at different locations (start, middle, and end). Each row corresponds to a specific scanning location, and the columns display the outcomes for one-pulse illumination, 30-pulse illumination, and the resulting cGAN denoising. The 2D raster scan experiment further reinforces these findings, with Fig. 5(a) illustrating the photobleaching progression at different cross-sections of the ICG-filled tube. The consistent photobleaching across the tube sections, apart from the initial starting point, shows that even slight shifts in imaging location result in notable reductions in signal amplitude. The uniform step size (100  μm) of the scan ensures that the results are comparable across sections, providing a reliable measure of the extent of photobleaching. Interestingly, the results suggest that ICG-water solutions are prone to photodamage even with relatively short intervals between cross-sectional scans. Although the volumetric scans and cross-sectional comparisons in Figs. 5(b) and 5(c) provide deeper insights into how photobleaching impacts 3D PA imaging, they also underscore the limitations posed by multipulse imaging in this context. In the one-pulse illumination, the PA signal was weaker but exhibited less cumulative photobleaching, preserving more structural information. However, in the 30-pulse illumination, although the signal was initially stronger, the cumulative effect of multiple laser pulses significantly exacerbated photobleaching, further diminishing the signal quality over time. This finding poses a challenge for multipulse imaging strategies: although they may initially offer higher SNR and better contrast, they can induce more pronounced photobleaching, leading to progressively poorer image quality, particularly in water-based ICG solutions.

Our denoising Pix2Pix-based cGAN DL model applied to one-pulse illumination images successfully generated a high SNR image without background noise [Fig. 3(c) third column]. This approach significantly reduced the scanning time by ∼25 to 30 times when compared with scans with averaging. Consequently, the impact of photobleaching was mitigated, and we obtained an image with satisfactory SNR and CNR. Although endogenous contrast agents are typically not affected by optical bleaching, exogenous contrast agents are prone to it over time. In future work, it will be important to explore strategies to mitigate photobleaching during PA imaging. These might include the development of more photostable contrast agents, optimizing laser parameters to minimize photodamage, or using adaptive imaging strategies that minimize exposure without compromising image quality. In addition, further research into the ability of DL models to correct photobleaching artifacts and their limitations in restoring true quantitative signal strength could offer pathways to improve post-processing techniques.

Most studies using DL for denoising PA images rely on numerically simulated data or *in vitro* phantoms, with limited application to *in vivo* datasets. When *in vivo* data are used, training and testing datasets often come from similar samples and the same imaging hardware, limiting generalizability and clinical relevance. In our study, training was conducted exclusively on metal wire phantom images from an LED-based AcousticX system with a 7 MHz transducer, whereas testing used AR-PAM images with a 25-MHz transducer. This introduced morphological and noise-profile differences between the datasets, providing a stringent test of the model’s generalization ability. By training on one platform and testing on another, we demonstrate that the DL model is not overfitted to the training platform’s characteristics. Instead, it generalizes across systems, accommodating diverse noise profiles, resolutions, and hardware configurations. This cross-platform validation highlights the model’s robustness and versatility, enabling its use across various clinical and research settings without retraining. Such flexibility enhances the utility of PA imaging for diverse experimental and biological applications, saving time and computational resources while maintaining high performance.

## 4 Conclusion

Photobleaching poses significant challenges in molecular PAI using exogenous dyes. Prolonged laser exposure degrades signal quality, limiting imaging duration and complicating quantitative analyses. Addressing these limitations, we developed a platform-flexible generative DL framework that leverages a custom loss function-based cGAN to enhance SNR in single-pulse acquired AR-PAM data after being trained with LED-illuminated images, effectively mitigating photobleaching risks. Our model is validated across two PA imaging systems, ensuring platform flexibility, where the model has adaptability to varying noise conditions while preserving critical image features. Unlike platform-independent models, our approach is optimized for specific systems while retaining the potential for broader applicability with minimal adaptation. The primary focus of this study was to develop and benchmark a computational framework for image denoising and signal enhancement using well-controlled *in vitro* and *ex vivo* datasets. These datasets, with their precise and reproducible conditions, were critical for establishing the reliability and efficacy of the proposed method, allowing for controlled experimentation and clear benchmarks for validation. We will integrate the proposed framework into real-time *in vivo* imaging workflows, addressing challenges such as physiological and motion-related artifacts that may impact performance evaluation. Our focus will be on validating the method under complex, dynamic conditions to further assess its adaptability and clinical utility. Other future work involves improving the image quality by integrating multimodal DL models combining PA and US data to fuse complementary information such as providing better anatomical context. We will look for self-supervised, few-shot or zero-shot DL techniques for better generalization with minimal labeled data.

## Supplementary Material

10.1117/1.JBO.30.S3. S34102.s01

## Data Availability

Materials are available upon request from the authors.
