# Supplementary material for: Reduction of photobleaching effects in photoacoustic imaging using noise agnostic, platform-flexible deep-learning methods
Source: J Biomed Opt. 2025 May 28;30(Suppl 3):S34102. doi: 10.1117/1.JBO.30.S3.S34102 (PMC12118878; doi:10.1117/1.JBO.30.S3.S34102)
Supplement: Supplementary file 1 [file JBO_030_S34102_SD001.docx]

Reduction of photobleaching effects in photoacoustic imaging using noise agnostic, platform-flexible deep-learning methods

Avijit Paul^a^, Christopher Nguyen^a^ , Tayyaba Hasan^b^, and Srivalleesha Mallidi^a,*^

aTufts University, Department of Biomedical Engineering, Medford, USA

bMassachusetts General Hospital, Harvard Medical School, Boston, USA


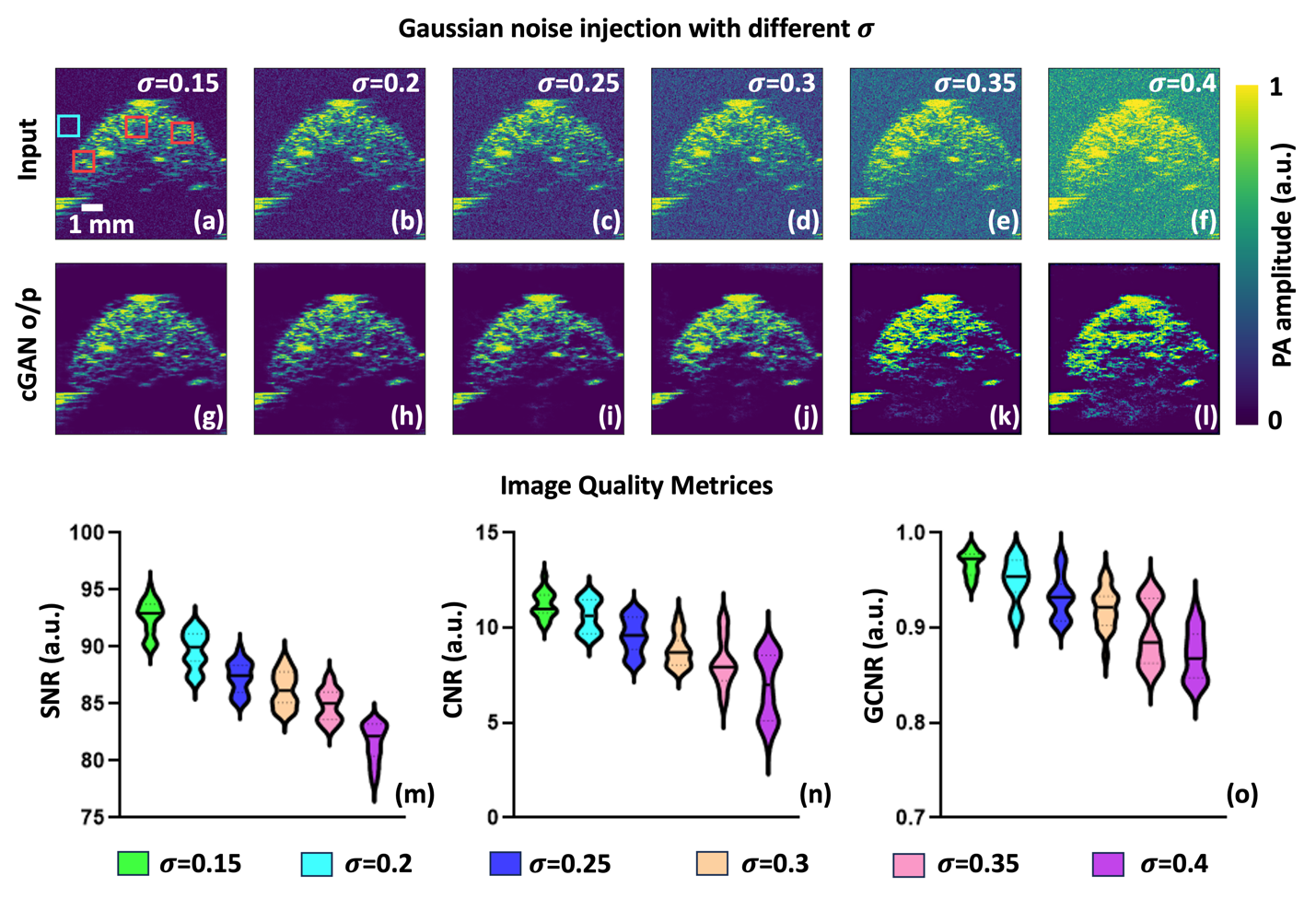


**Fig. S1 Validation of the model's flexibility and robustness to diverse Gaussian noise conditions.**
(a-l) Denoising performance on AR-PAM high-pulse-averaged data with synthetically injected Gaussian noise with different variance mentioned at the top right corner of each input image. (i-k) Violin plots of image quality metrics (e.g., SNR, CNR, and GCNR) across datasets with varying noise profiles, showcasing the model's noise-invariance capabilities. The three sample PA signal regions are denoted by red boxes and cyan colored box denotes the background region.

**Table ST1** Image quality metrices for different loss function combinations in DL denoising methods.

| Loss1 = gan_loss + (LAMBDA * l1_loss) |
| --- |
| Loss2 = gan_loss + (LAMBDA * l2_loss) |
| Loss3 = gan_loss + (LAMBDA * huber_loss) |
| Loss4 = gan_loss + (LAMBDA * l1_loss) + (LAMBDA * ssim_loss) |
| Loss5 = gan_loss + (LAMBDA * l2_loss) + (LAMBDA * ssim_loss) |
| **Loss6 = gan_loss + (LAMBDA * huber_loss) + (LAMBDA * ssim_loss)** |
| Loss7 = gan_loss + (LAMBDA * l1_loss) + (LAMBDA * ssim_loss) + psnr_loss |
| Loss8 = gan_loss + (LAMBDA * l2_loss) + (LAMBDA * ssim_loss) + psnr_loss |
| Loss9 = gan_loss + (LAMBDA * huber_loss) + (LAMBDA * ssim_loss) + psnr_loss |

| **Imaging Process** | | **Adam** | | **Nadam** | | **Rectified Adam** | **AdaBelief** | **Yogi** |
| --- | --- | --- | --- | --- | --- | --- | --- | --- |
|  |  | |  | | ***SNR*** | | | |
| Loss1 | | 90.4912 | | 90.2746 | | 91.8419 | 90.6841 | 90.0412 |
| Loss2 | | 89.7996 | | 90.7021 | | 91.2015 | 90.6112 | 89.4537 |
| Loss3 | | 91.6012 | | 89.4703 | | 90.4208 | 91.3017 | 88.8435 |
| Loss4 | | 92.0879 | | 91.1905 | | 91.0167 | 91.9346 | 92.0674 |
| Loss5 | | 92.4734 | | 91.5787 | | 91.1078 | 90.8915 | 91.6766 |
| **Loss6** | | **92.7763** | | 92.1605 | | 91.5933 | 91.6106 | 92.0026 |
| Loss7 | | 91.8397 | | 91.0878 | | 91.8016 | 92.1098 | 91.5655 |
| Loss8 | | 85.1402 | | 91.4452 | | 92.4301 | 87.4151 | 92.1191 |
| Loss9 | | 91.3289 | | 91.6907 | | 91.8265 | 92.0042 | 90.5467 |
|  | |  | |  | |  |  |  |
|  |  | |  | | ***CNR*** | | | |
| Loss1 | | 8.8867 | | 8.9509 | | 8.3934 | 8.3099 | 8.7938 |
| Loss2 | | 8.6193 | | 8.3427 | | 8.7842 | 8.9503 | 8.4774 |
| Loss3 | | 8.9199 | | 8.9306 | | 8.4955 | 8.1158 | 8.3512 |
| Loss4 | | 9.3112 | | 8.4877 | | 8.5729 | 8.7279 | 8.6174 |
| Loss5 | | 9.2448 | | 8.6594 | | 9.1462 | 8. 5495 | 9. 0468 |
| **Loss6** | | **9.9018** | | 9.2143 | | 8.8446 | 9.0054 | 8.6174 |
| Loss7 | | 8.9817 | | 8.5974 | | 8.5325 | 8.4429 | 9.0087 |
| Loss8 | | 7.8914 | | 8.0018 | | 8.1881 | 8.0952 | 8.1193 |
| Loss9 | | 8.9746 | | 8.8725 | | 9.0147 | 8.7853 | 8.9517 |
|  | |  | |  | |  |  |  |

**Table ST2** Image quality metrices for delta variations of Huber loss in DL denoising methods (with Adam).

| **Huber Parameter** | **SNR** | **CNR** |
| --- | --- | --- |
| Delta = 0.5 | 91.0941 | 9.0545 |
| **Delta = 1** | **92.7763** | **9.9018** |
| Delta = 1.5 | 90.8961 | 8.5641 |
|  |  |  |

**Table ST3** Image quality metrices for architectural changes in DL denoising methods (with Adam).

| **Architectural changes** | **SNR** | **CNR** |
| --- | --- | --- |
| **RELU + Batch Normalization** | **92.7763** | **9.9018** |
| RELU + Weight Normalization | 92.4657 | 9.4507 |
| RELU + Spectral Normalization | 92.1985 | 9.0085 |
| LeakyRELU + Batch Normalization | 92.3101 | 8.9874 |
| LeakyRELU + Weight Normalization | 91.7366 | 9.0142 |
| LeakyRELU + Spectral Normalization | 92.0527 | 9.0187 |
|  |  |  |
